# Exploring the Healthcare Seeking Behavior of Medical Aid Beneficiaries Who Overutilize Healthcare Services: A Qualitative Descriptive Study

**DOI:** 10.3390/ijerph16142485

**Published:** 2019-07-12

**Authors:** Jeonghyun Cho, Kyungin Jeong, Samsook Kim, Hyejin Kim

**Affiliations:** 1 Department of Nursing, Inje University, Busan 47392, Korea; 2 Department of Nursing, Daedong College, Busan 46270, Korea; 3 Red Cross College of Nursing, Chung-Ang University, Seoul 06974, Korea

**Keywords:** health seeking behavior, Medical Aid, medical overuse, qualitative research

## Abstract

We explored the healthcare seeking behavior of Medical Aid (MA) beneficiaries who over-utilize healthcare services in South Korea. We employed a qualitative descriptive study using semi-structured interviews with fifteen community-dwelling MA beneficiaries—who were categorized as over-utilizing healthcare services—and conventional content analysis. Four categories emerged: having unmet healthcare needs, wandering in search of effective healthcare services, routinizing their use of healthcare services, and being concerned about benefit restrictions due to their healthcare overutilization. Overall, these categories indicate participants’ behaviors used to fill the gap between their high healthcare needs and restricted MA benefits. The findings provide a foundation for healthcare providers, policymakers, researchers, and MA beneficiaries to discuss how to better address beneficiaries’ healthcare needs while preventing healthcare overutilization patterns. Effective and innovative interventions that target MA beneficiaries and their case managers are necessary to improve beneficiaries’ quality of life.

## 1. Introduction

Since 1977, the Medical Aid (MA) program has been operated by the government in South Korea as a national public assistance program for low-income people who are impoverished or unable to sustain their livelihoods on their own [1]. It provides minimum medical security for low-income families [2], which is similar to Medicaid in the U.S., the National Health Service Low Income Scheme in U.K., and the medical assistance program (i.e., Hilfen zur Gesundheit) in Germany [3]. The MA program covers healthcare expenses for a wide spectrum of treatments, diagnostic tests, and medications for MA beneficiaries [1]. However, healthcare overutilization has been observed and is related to the low financial burden for MA beneficiaries [4,5]. There were about 1.5 million MA beneficiaries in 2017, accounting for 2.8% of the total population in South Korea. In the same year, the total healthcare costs for these MA beneficiaries were approximately $6.3 billion, indicating a cost of $4200 per person and a 5.2% increase from the previous year [6]. Considering that a National Health Insurance (NHI) enrollee (97.6% of the total population) expensed an average of $1190 in medical costs [6], multi-faceted interventions are necessary to address healthcare overutilization of MA beneficiaries.

Most MA beneficiaries are older adults and have multiple uncontrolled conditions and diseases. Due to their financial hardships or poverty, they are unable to receive appropriate, timely treatments, leading to health deterioration [7]. Once they become eligible to receive medical benefits under the MA program, they are given greater access to healthcare services. However, since they have high healthcare demands due to increased age, multiple chronic conditions, and high disease severity, frequent use of healthcare services by these patients is common and often inevitable [2,5]. Case managers are responsible for assessing the healthcare needs of MA beneficiaries and providing health counseling and education to improve beneficiaries’ quality of life and health-related behaviors. However, each case manager is responsible for overseeing the needs of more than 100 beneficiaries per year [1], these beneficiaries’ medical and informational demands may not be adequately addressed.

To date, studies have evaluated national healthcare-related policies mostly by focusing on assessing healthcare utilization and expenditures. The quality of healthcare services and the healthcare seeking behaviors of MA beneficiaries have rarely been investigated [8]. Although a few studies have investigated the experiences of MA beneficiaries [7,9], no studies have focused on the experiences of MA beneficiaries who over-utilize healthcare services. Such studies are vital for the development of a person-centered intervention program to address MA beneficiaries’ healthcare needs while avoiding unnecessary healthcare expenditures. Therefore, the purpose of this study was to explore healthcare seeking behaviors from the perspective of MA beneficiaries who over-utilize healthcare services in South Korea. We focused on these beneficiaries’ healthcare utilization processes. In this study, beneficiaries who over-utilize healthcare services were those categorized in high-risk groups on the annual assessment of MA case management (i.e., those who over-utilized medical benefits relative to their diseases, such as “hospital shopping,” drug overuse, or duplicate prescription) and those categorized in intense management groups (i.e., those whose behaviors of healthcare service use could not be changed by short-term case management but required consistent management and intervention) [1]. This study contributes to the development of effective and efficient healthcare models for MA beneficiaries.

## 2. Materials and Methods

We used a qualitative descriptive approach with in-depth, semi-structured interviews and conventional content analysis. Qualitative description was chosen because it allows us to “stay closer to the data and to the surface of words and events” than other qualitative traditions [10] (p. 336) to describe the healthcare seeking process from the perspective of MA beneficiaries who over-utilize healthcare services. This study was approved by the Inje University Institutional Review Board (INJE 2017-06-004-001), and all procedures complied with the institution’s ethics protocol. Participant recruitment and data collection occurred between 21 July 2017 and 1 March 2018. We reported all procedures and findings using the consolidated criteria for reporting qualitative research (COREQ) [11].

### 2.1. Participants and Setting

MA beneficiaries residing in four districts within B City, South Korea were recruited using purposive sampling. Participants were beneficiaries (1) who had been receiving MA benefits for at least three years, (2) in high-risk groups on the latest annual assessment of MA case management or in intense management groups [1], and (3) who were not cognitively impaired and were willing to participate. We selected participants who had been receiving the MA benefits for at least three years for two reasons. First, high-risk and intensive management groups, indicating beneficiaries who over-utilize healthcare, were determined based on healthcare utilization data from the prior year. Second, newer beneficiaries may still be learning about the MA program and are at a greater risk for potentially inappropriate use of MA services. MA beneficiaries who were selected based on different laws (e.g., national merit recipients, North Korean refugees, etc.) were excluded since they receive benefits through diverse mechanisms.

Three MA case managers identified MA beneficiaries who met the inclusion criteria and secured beneficiaries’ permission to share their contact information with the researchers. The researchers contacted each participant to explain the study purpose and process, addressed their concerns and questions, and received written informed consent from those who agreed to participate.

### 2.2. Data Collection

We conducted individual, in-depth, semi-structured interviews with participants using an interview guide that included open-ended questions about their health status, healthcare service use and rationale for the use, level of satisfaction with the use of the MA program, and efforts to maintain health. Interview questions included: “How have you been using healthcare services (e.g., visiting hospitals, medications)?”; “How did you learn about the hospitals you currently visit?”; “How do you feel about the healthcare services you are currently using?”; and “What kinds of efforts have you made to stay as healthy as possible?” In this study, the term “hospital” referred to inpatient and outpatient healthcare settings. Interviews were conducted by three authors (J.C., K.J., and S.K.). To ensure interview consistency, two of the three authors initially conducted paired interviews for the first three participants. The three authors then listened to the recordings together and adjusted their interviewing styles to use a similar approach for the remaining interviews. Nonverbal clues (e.g., participants’ facial expressions), interview situations, and researchers’ emotions were documented immediately after each interview. Interviews were conducted until no new information was yielded. After reviewing the interview with the fourteenth participant, we noted that the content was redundant, and we proceeded with a final interview (*n* = 15) to confirm data saturation. Interviews took approximately 40 min to 1.5 h and were conducted in Korean at participants’ homes.

Interviews were audio-recorded and transcribed verbatim. The data collectors reviewed the transcripts and removed participants’ personal identifiers to maintain confidentiality. All text-data were managed using NVivo 11 (QSR International, Burlington, MA, USA).

### 2.3. Data Analysis

Data were analyzed using the conventional (or inductive) content analytic method [12,13] to describe the healthcare seeking process of MA beneficiaries who over-utilized healthcare services. Three authors (J.C., K.J., and S.K.). participated in the analytic process and two of them were assigned to each transcript. The authors read the transcript multiple times to gain a general sense of the data, which was followed by open coding. They coded transcripts line-by-line independently and then met to compare and reconcile their codes. Unresolved discrepancies between the two authors were discussed within the research team to reach an agreement. Then, the authors collapsed relevant codes into subcategories, which were further grouped into categories. Throughout the entire analysis process, the research team continually discussed and refined codes and categories. For the presentation of the findings, the research team selected quotes that represented the developed subcategories and categories.

### 2.4. Rigor

The trustworthiness of the study was maintained through a qualitative audit and peer debriefing [14,15]. A qualitative expert who did not participate in the initial analysis (H.K.) reviewed the analytic process and offered suggestions to improve the clarity of the results. Preliminary findings were also discussed with case managers who were not involved in this study to obtain experts’ opinions about the findings and implications for practice. The case managers agreed with the findings; thus, we did not make any changes to the findings. Once categories and supporting quotations were determined, they were translated into English by a professional translator. Two authors (J.C. and H.K.) then reviewed the translated categories and quotations for their accuracy with respect to the conveyed meanings.

## 3. Results

### 3.1. Participants’ Characteristics

Fifteen MA beneficiaries participated in this study (nine women, six men; median age = 71 years; range = 51−83 years). Six participants had completed high school, three had graduated from a technical college, two had completed elementary school, and four had received no formal education. Thirteen MA beneficiaries were Class 1 (i.e., National Basic Livelihood Security beneficiaries who were unable to work; had tuberculosis, rare or intractable disorders, or severe diseases; or were residing in assisted living facilities operated by the local welfare department or homeless patients) and two were Class 2 (i.e., National Basic Livelihood Security beneficiaries who did not belong to Class 1). The duration of coverage under the MA program ranged from 4 to 28 years (mean duration = 13.6 ± 10.7 years). Participants lived with three to eight diseases: The most common were hypertension, degenerative arthritis, spinal stenosis, and depression.

### 3.2. MA Beneficiaries’ Healthcare Seeking Process

**Figure 1:**
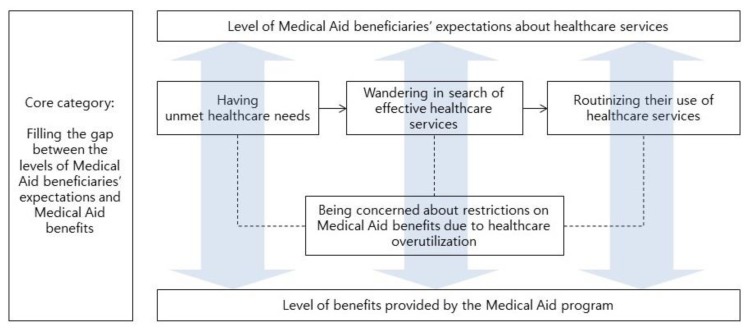
The healthcare seeking process of Medical Aid beneficiaries.

Participants over-utilized healthcare services to fill the gap between the levels of their expectations about healthcare services and benefits provided by the MA program (see Figure 1). While the level of participants’ expectations for recovery from illnesses and symptom relief was high, the healthcare benefits provided by the MA programs did not meet participants’ expectations. In other words, they had unmet healthcare needs. To have their healthcare needs met, participants continued to seek effective healthcare services. This continuous search for healthcare services and visits to hospitals became a daily routine. While continually seeking healthcare, participants were aware of and concerned about the possibility of having restrictions on their healthcare use due to their continuous overutilization. Despite this awareness, they continued to visit various hospitals and did not reduce their use of healthcare services.

#### 3.2.1. Having Unmet Healthcare Needs

Some participants could not undergo necessary tests or treatments due to their high costs and the high deductibles imposed on them. One participant articulated, “*The [MA] benefits have certainly been useful for medical examinations and medication costs. However, most tests that my doctors recommend when I am sick are not covered by the benefits*” (P4). They also complained of medications prescribed for their acute and chronic illnesses. As their pain and symptoms remained, even though they took the medications as prescribed, they perceived that the medications allowed within the MA benefits were less effective than those covered by NHI. Consequently, they expressed disappointment, frustration, and helplessness:

“*I heard that there’s a difference between the medication that you pay for and the medication that is covered by the MA benefits, even if it’s the same. I have heard that medications given to MA beneficiaries are the least potent. I think that’s why I don’t get any better after taking these medications. It is unfair! Higher potent medications are not given to us, MA beneficiaries, because they are expensive. I, as a beneficiary, think this is absolutely unfair!*” (P1)

#### 3.2.2. Wandering in Search of Effective Healthcare Services

Participants explained that, when they first received healthcare benefits under the MA program, they thought they were receiving a tremendous amount of benefits since they had to pay little-to-no fees for hospital examinations and pharmacy services:

“*Initially, I was skeptical as to whether they would really give me medical benefits. Once I saw a huge reduction in my medication costs, I felt the advantage of having the MA benefits. I was thinking, ‘Is this true? Would it change in the future?*” (P4)

As participants realized that they could use these services without limit within the scope of the benefits, they tended to visit numerous hospitals more frequently to seek relief from bothersome symptoms. However, participants reported difficulties finding effective healthcare services, thus continuously switching hospitals or services:

“*I experienced no improvement in symptoms regardless of which hospital I visited. Treatments were of no use, and that’s why I keep switching from one hospital to another. A doctor once told me my disease would not be cured. I feel frustrated, so I keep changing hospitals because of this frustration.*” (P7)

While seeking information to relieve pain and symptoms, participants received information from their neighbors and friends about which hospitals were effective for treating those symptoms: “*Every time someone would recommend a hospital, I would immediately go to that hospital. Neighbors would recommend hospitals to me… You know, people say old grannies know best about hospitals*” (P14).

#### 3.2.3. Routinizing Their Use of Healthcare Services

Although participants were aware that their health status did not improve even after visiting numerous hospitals, they continued to visit those hospitals. This was because they believed that their health status would deteriorate if they skipped their daily visit. One participant stated, “*After a few days of not going to the hospital, my body feels stiff. I don’t want my health to worsen*” (P6). Another participant also reported, “*I am still in pain after receiving shots. The pain remains even after physiotherapy, but I still go to the hospital because the pain might become worse if I stop going*” (P5). Thus, they visited at least one hospital each day, which became their daily routine. For example, some participants visited various hospitals all day to receive healthcare services:

“*I wake up in the morning, eat breakfast, go to a hospital around 11 am or noon, receive physiotherapy, and then go to an otolaryngology clinic because my throat hurts. Sometimes, the sun is already setting after I’ve visited hospitals.*” (P10)

This habitual visit pattern made them view hospitals as their second homes and even consider going to the hospital as exercise.

“*I go to the hospital every day, so it feels like going home now. I go there without much thought, and then I return home. I can’t go when the hospital is closed, but when it is open, I go there to receive physiotherapy, get shots, and so forth. There’s a clinic 200 m away. I walk up to the hospital and consider it exercise.*” (P5)

#### 3.2.4. Being Concerned about Restrictions on MA Benefits Due to Healthcare Overutilization

Participants were aware of their frequent visits to hospitals and felt the need to reduce their use of healthcare services:

“*I just have to bear the pain. I feel embarrassed and sorry every time I receive a letter from the hospital. But I am trying as much as possible not to go to hospitals. I don’t want to add more burdens to my country. I keep having this thought.*” (P2)

However, because they were in pain, they described having no choice but to go to hospitals to receive healthcare services. In addition, due to their multiple illnesses, they were consistently being prescribed medications at various hospitals. As they visited hospitals frequently and received medications for each illness, the number of days that they used MA benefits increased (in South Korea, the healthcare utilization under the MA program is calculated in terms of the number of days using services). Therefore, they were concerned and often anxious about possible MA benefit restrictions, such as having a designated hospital (limited freedom in hospital selection) if they continued to use these services as much as they did:

“*I am super worried about hospital designation. I don’t think I’ll be able to stand it if I get a designated hospital. That’s why every time I come back from a hospital, I record the date of the visit, prescriptions, and everything.*” (P2)

One other participant also described her concerns and anxiety about the potential MA benefit restriction: “*My heart sinks every time I get a phone call from the district office. Just anything—letters or phone calls—makes my heart sink*” (P11).

## 4. Discussion

This qualitative descriptive study was conducted to describe the healthcare seeking process of MA beneficiaries who over-utilized healthcare services in South Korea. By analyzing interviews with 15 MA beneficiaries who were categorized as over-utilizing their healthcare benefits, we presented a four-category model. This included having unmet healthcare needs, wandering in search of effective healthcare services, routinizing their use of healthcare services, and being concerned about restrictions on MA benefits due to healthcare overutilization. Overall, this model depicted participants’ behaviors used to fill the gap between their high healthcare needs and the healthcare benefits allowed by the MA program.

Participants perceived that their healthcare needs, such as pain management requiring more advanced tests and procedures, were unmet, primarily due to the limited benefits allowed for them. Shon and Kim [5] found that MA beneficiaries were more likely to have high healthcare needs due to their increased disease severity, low income, and poor perceived health status. Notably, the healthcare benefits allowed for these beneficiaries did not meet their high healthcare needs [1]. Our participants also stated that the MA benefits did not cover advanced diagnostic tests, therapeutic procedures, or expensive medications that were often recommended by their healthcare providers. Due to their poor financial status; however, these beneficiaries could not afford such out-of-pocket healthcare services. Instead, they kept searching for healthcare services that could meet their needs within the scope of the guaranteed benefits and increased the frequency of using healthcare services covered by the benefits, regardless of their dissatisfaction. In recent studies [4,16], the number of MA beneficiaries using outpatient healthcare services was higher than that of the NHI enrollees using such outpatient services.

Our findings show that this “hospital shopping” and frequent hospital-visit behaviors became routine, which was reinforced by participants’ concerns about worsening symptoms if they did not visit hospitals. Once health behaviors become routine, they become difficult to reverse since health behaviors have a natural resistance to change [17]. Pender [18] showed that health behaviors are also affected by physiological (e.g., depression, uncertainty) and sociocultural factors (e.g., race, social support). Our finding that participants kept their healthcare utilization behaviors despite their concerns about potential benefit restrictions related to their healthcare overutilization also indicates that it is difficult to change beneficiaries’ health behaviors. This suggests the need for individualized programs targeting such psychological and sociocultural factors associated with these health behaviors.

To understand MA beneficiaries’ healthcare overutilization, more attention should be given to the quality of healthcare services that MA beneficiaries receive. To date, studies have evaluated the impact of MA benefits using quantitative indices, such as the number of MA beneficiaries [19], healthcare-service days [4,16,20], and healthcare expenses [4,16,20]. Although the MA program provides its beneficiaries with basic healthcare security, little information exists regarding the quality of received healthcare services. It is possible that these beneficiaries might have received less effective healthcare services for their health problems. In a recent study [8], MA beneficiaries had longer hospital stays and more preventable hospitalizations than did NHI enrollees, indicating that MA beneficiaries were less likely to receive timely and adequate healthcare services despite their high rates of service utilization. Thus, more research is necessary to evaluate the quality of healthcare services that MA beneficiaries receive (e.g., beneficiaries’ satisfaction with healthcare services, health-related quality of life, perceived health status), which also requires the development of robust assessment tools.

The role of case managers is also critical for MA beneficiaries’ improved health status, health behaviors, and quality of life [21,22,23]. In South Korea, case management has been implemented to improve the quality of life and health status of MA beneficiaries, while ensuring that they make reasonable use of healthcare services [1]. Previous studies have shown that case management is effective in healthcare continuity and for expenditure reductions [24,25]. Especially for community-based case management, it is vital for case managers to interact within the community healthcare service network. This process should involve arranging and coordinating healthcare services for MA beneficiaries in a supportive, efficient, and cost-effective manner [26]. To provide such supportive, efficient, and cost-effective care to these beneficiaries, case managers need to conduct comprehensive assessment of these beneficiaries’ health conditions, including individuals’ physical, psychological, social, environmental, and spiritual health [27]. They should then identify the main healthcare needs of each beneficiary, navigate the most appropriate healthcare services for each person, and follow up with them about the resulting effects. If a negligible effect is observed, case managers should advise beneficiaries to consult their healthcare providers rather than go “hospital shopping.” However, little is known about the reality of case managers’ practices with MA beneficiaries. Furthermore, few studies have examined the effects of interventions that target case managers, which calls for robust, theory-driven intervention studies that aim to improve case managers’ care coordination and delivery.

In this study, participants did not report their relationships with their case managers or healthcare providers in relation to their healthcare seeking behaviors. This may indicate that the participants might not have discussed their health conditions, concerns, and healthcare services with case managers when seeking and selecting healthcare services (e.g., hospitals, medication). Moreover, participants might not have received appropriate, sufficient support from case managers regarding the management of health conditions and the appropriate use of healthcare services. It is also possible that information from their friends or neighbors had a stronger impact on their selection and utilization of healthcare services than did their relationships with case managers or healthcare providers. To manage their health conditions in an effective, efficient way, MA beneficiaries require proper healthcare information about their health conditions on an ongoing basis, through various modalities, such as individualized in-person or telephone consultations and group sessions. It is vital for case managers to address beneficiaries’ informational needs for their health care and provide useful community resources. Furthermore, more community-based programs—such as group-based learning or activity sessions related to common health problems in this population—may provide ample opportunities for beneficiaries to build the knowledge and skills necessary for better health management [28,29]. More research is needed to develop and test diverse, community-based, healthcare-related educational or activity programs targeting MA beneficiaries to improve their quality of life.

While this study provides unique and beneficial information, several limitations should be discussed. Because data were collected from MA beneficiaries categorized as an overutilization group in a single province in South Korea, the findings may not apply to those outside this category or region. In addition, we did not seek opinions from various stakeholders such as case managers, healthcare providers, and policymakers, which are necessary to have a more comprehensive understanding of MA beneficiaries’ healthcare utilization. Despite these limitations, this novel study describes MA beneficiaries’ healthcare seeking behaviors from their perspectives. Future research needs to illustrate MA beneficiaries’ healthcare utilization from varied perspectives; examine factors that affect the process of such healthcare utilization; and develop effective, innovative, person-centered case-management programs for this population.

## 5. Conclusions

This study explored MA beneficiaries’ healthcare overutilization behaviors. Our findings provide a foundation for healthcare providers, policymakers, researchers, MA beneficiaries, and other stakeholders to discuss how to better address MA beneficiaries’ healthcare needs and prevent healthcare overutilization. To enhance beneficiaries’ quality of life and reduce healthcare expenditures, there is an urgent need for effective and innovative interventions (e.g., educational programs or resources) that target the beneficiaries and their case managers.
